# Safety of 12 core transrectal ultrasound guided prostate biopsy in patients on aspirin

**DOI:** 10.1590/S1677-5538.IBJU.2015.0053

**Published:** 2015

**Authors:** Pawan Vasudeva, Niraj Kumar, Anup Kumar, Harbinder Singh, Gaurav Kumar

**Affiliations:** 1 Department of Urology, V.M. Medical College and Safdarjang Hospital, New Delhi 110029, India

**Keywords:** Prostate, Biopsy, Aspirin, Hemorrhage

## Abstract

**Objective:**

To prospectively assess safety outcome of TRUS guided prostate biopsy in patients taking low dose aspirin.

**Materials and methods:**

Consecutive patients, who were planned for 12 core TRUS guided prostate biopsy and satisfied eligibility criteria, were included in the study and divided into two Groups: Group A: patients on aspirin during biopsy, Group B: patients not on aspirin during biopsy, including patients in whom aspirin was stopped prior to the biopsy. Parameters included for statistical analysis were: age, serum prostate specific antigen (PSA), prostate volume, hemoglobin (Hb %), number of hematuria episodes, number of patient reporting hematuria, hematuria requiring intervention, number of patient reporting hematospermia and number of patient reporting rectal bleeding.

**Results:**

Of 681 eligible patients, Group A and B had 191 and 490 patients respectively. The mean age, prostate volume, serum PSA and pre-biopsy hemoglobin were similar in both Groups with no significant differences noted between them. None of the post-biopsy complications, including number of hematuria episodes (p=0.83), number of patients reporting hematuria (p=0.55), number of patients reporting hematospermia (p=0.36) and number of patients reporting rectal bleeding (p=0.65), were significantly different between Groups A and B respectively. None of the hemorrhagic complication in either group required intervention and were self limiting.

**Conclusion:**

Continuing low dose aspirin during TRUS guided prostate biopsy neither alters the minor bleeding episodes nor causes major bleeding complication. So, discontinuation of low dose aspirin prior to TRUS guided prostate biopsy is not required.

## INTRODUCTION

Growing life expectancy and resultant ageing population, along with increasing awareness and use of serum prostate specific antigen (PSA) for prostate cancer screening led to increase in transrectal ultrasound (TRUS) guided prostate biopsy, a gold standard procedure for histopathological diagnosis of prostate cancer, in urological practice.(1–3) 10-12 systematic cores for initial diagnosis have been suggested by European Association of Urology (EAU) 2014 guidelines (level of evidence 2a, grade of recommendation B).(4) A high proportion of patients requiring TRUS guided prostate biopsy for diagnosis of prostate cancer are on medications like aspirin, warfarin, etc. for associated co-morbidities (3). With 12 core TRUS guided prostate biopsy, although minor and self limiting, hemorrhagic complications like hematuria, hematospermia and rectal bleeding were reported in 33-39%, 12-36% and 14-27%, respectively (3, 5, 6).

Literature regarding TRUS guided prostate biopsy in aspirin users report variable results, some observed no difference in bleeding complications, while others reported higher rate of minor bleeding complications (1, 6–8). In this study, we intended to prospectively assess safety outcome of TRUS guided prostate biopsy in low dose aspirin users.

## MATERIALS AND METHODS

This prospective study was performed in our hospital in the period between April 2011 and November 2014. Indications for biopsy were serum PSA>4ng/mL and/or abnormal digital rectal examination. Consecutive patients, planned for 12 core TRUS guided prostate biopsy, were included in the study, whereas patients with: a) History of bleeding disorder; b) Patient on anticoagulant other than aspirin; c) <or>12 biopsy cores and; d) patients who did not sign informed consent, were excluded. Patients were non-randomly divided into two Groups: Group A) patients on low dose (75mg per day) aspirin during biopsy; Group B) patients not on aspirin during biopsy, including patients in whom aspirin was stopped prior to the biopsy.

Biopsy procedure: All patients got proctoclysis enema in the morning of biopsy. Ciprofloxacin 500mg orally was given 1 hour prior to biopsy and continued twice daily for 5 days. For analgesia, either 2% lignocaine jelly, eutectic mixture of lignocaine and prilocaine (EMLA) cream or periprostatic nerve block was used depending on patient choice. TRUS was performed in left lateral decubitus position using 7.5Hz bi-planar probe to assess prostate volume and then 12-core TRUS guided prostate biopsy was done using 18G disposable biopsy gun. Each biopsy core was collected in separate jar and sent for histopathological examination. Patients were observed for 2 hours post procedure and then sent home with advice to report about complications, if any. Patients were followed up at three weeks with biopsy report and query was made regarding complications.

### 

#### Data included for analysis included

##### Before biopsy

Age, serum prostate specific antigen (PSA), prostate volume, hemoglobin (Hb %), platelet count, serum creatinine.

##### After biopsy

Number of hematuria episodes, number of patient reporting hematuria, hematuria requiring intervention, number of patient reporting hematospermia and number of patient reporting rectal bleeding.

### Statistical Analysis

Recorded study parameters were arranged on a Microsoft excel spreadsheet (Microsoft, Seattle, WA USA) and SPSS (IBM SPSS Statistics 21.0; IBM SPSS, 2012) software package was used for analysis. The continuous data were expressed as mean±standard deviation and analyzed by Student t-test whereas categorical data were expressed as number/percentages and analyzed by Fisher exact tests. P values<0.05 were considered statistically significant.

## RESULTS

Of 783 TRUS guided prostate biopsy during the study period, 681 satisfied eligibility criteria and data of these patients were analyzed for the study. Of these, 191 patients were receiving aspirin during the biopsy, while in rest of 490 patients either the aspirin was stopped prior to biopsy or were not receiving aspirin. Table-1 summarized the baseline characteristics of patients in both Groups. The mean age (67.25 vs. 66.97, p=0.66), prostate volume (61.14 vs. 62.51, p=0.41), serum PSA (31.25 vs. 29.70, p=0.66), pre-biopsy hemoglobin (12.92 vs. 12.78, p=0.15), platelet count (137.94 vs. 142.67, p=0.27) and serum creatinine (1.21 vs. 1.17, p=0.26) were similar in the two Groups (A vs. B) with no significant differences noted between them. Although Group A had significantly higher number of patients with cardiovascular disease (182 vs. 68, P<0.0001) compared to Group B, other co-morbidities including cerebrovascular disease (23 vs. 40, p=0.18), diabetes (70 vs. 160, p=0.50) and chronic obstructive pulmonary diseases (30 vs 56, p=0.20) had similar distribution among the patients of both Groups (A vs. B).

**Table 1:** Baseline characteristics.

	Group A (n=191)	Group B (n=490)	P value*
Age (years)	67.25±7.44	66.97±7.72	0.66
Prostate Volume (mL)	61.14±18.96	62.51±20.09	0.41
Serum PSA (ng/mL)	31.25±43.82	29.70±40.87	0.66
Hb (gm %)	12.92±1.04	12.78±1.13	0.15
Platelet Count (x10^3^ per microlitre)	137.94±46.34	142.67±52.20	0.27
Serum Creatinine (mg/dL)	1.21±0.45	1.17±0.38	0.26

* Student t-test.

Table-2 summarizes the post-biopsy complications. None of the complications, including the mean number of hematuria episodes (1.87 vs. 1.83, p=0.83), number of patient reporting hematuria (86 vs 247, p=0.55), number of patient reporting hematospermia (21 vs. 40, p=0.36) and number of patient reporting rectal bleeding (29 vs. 65, p=0.65), were significantly different between Groups A and B respectively. None of the hemorrhagic complication in either Group required intervention and were self-limited.

**Table 2:** post-biopsy complications.

	Group A (n=191)	Group B (n=49 0)	P value*
Number of hematuria episodes	1.87±1.22	1.83±1.48	0.83#
Number of patient reporting hematuria	86 (45.02%)	247 (50.40%)	0.55
Hematuria requiring intervention	0	0	
Number of patient reporting hematospermia	21 (10.99%)	40 (8.16%)	0.36
Number of patient reporting rectal bleeding	29 (15.18%)	65 (13.26%)	0.65

# Student t-test,

* Fisher exact tests

**Figure 1:**
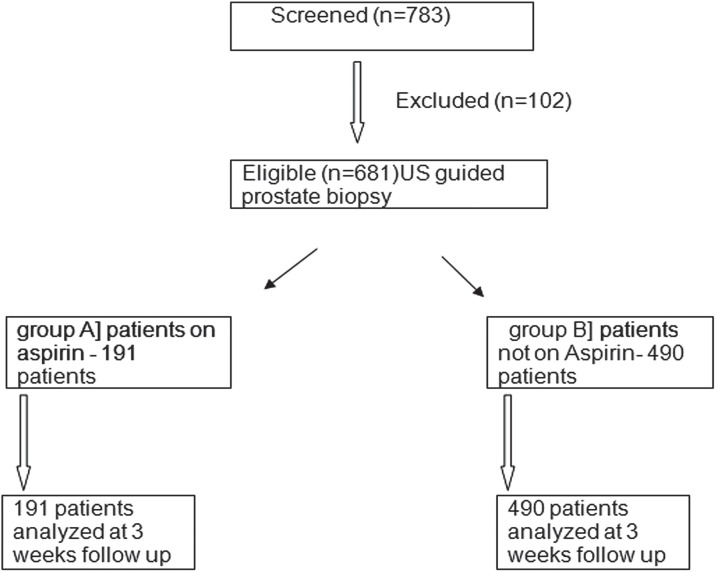
Allocation and dispersion of patients.

## DISCUSSION

Adoption of serum PSA screening for prostate cancer resulted in high number of TRUS guided prostate biopsies (6). 12 core prostate biopsy, with addition of laterally directed cores, improved cancer detection rate with complications not significantly different from sextant biopsies (9). There are no clear guidelines regarding TRUS guided prostate biopsy in patients on aspirin. At our center, we did not stop aspirin routinely prior to biopsy unless advised specifically by cardiologist because rebound thromboembolic complication following aspirin discontinuation is a known risk. It is suggested that low-dose aspirin should be discontinued only if bleeding risk outweigh the cardiovascular risk of aspirin discontinuation (10–12). However, Connor SEJ and Wingate JP in a survey among practicing radiologists and urologists observed that 52% of radiologists and 27% of urologists stopped aspirin prior prostatic biopsy (13).

Chowdhury R et al., in a prospective study involving 216 patients on low dose aspirin, observed that between aspirin users and non-users, hematuria, (33.8% vs. 37%), rectal bleeding (14.4% vs. 11.5%) and hematospermia (12% vs. 13.8%) rates did not vary significantly. They concluded that cessation of low dose aspirin prior to biopsy is not necessary (3). Halliwell O et al., in a prospective assessment of 1022 aspirin users and non-users patients, observed higher hematuria (72 vs. 61%, p<0.001), duration of hematuria (4.05 vs. 2.85 days, p<0.01), rectal bleeding (21 vs. 13%) among aspirin users. Hematospermia was not significantly different between both Groups. They also observed that although bleeding rates were higher with aspirin use, no patient required intervention for bleeding complications (1). Giannarini G et al. in a prospective trial including 200 patients observed that low dose aspirin did not increase bleeding rates but it prolonged the duration of hematuria and rectal bleeding (8). Carmignani L et al., in meta-analysis of TRUS guided prostate biopsy in patients taking aspirin, included 3218 patients and observed that compared to control, aspirin users have significantly higher rate of mild hematuria (p=0.001), whereas rate of rectal bleeding (p=0.33) and hematospermia (p=0.24) were not significantly altered. They came to conclusion that stopping aspirin prior to TRUS guided prostate biopsy is not necessary since it did not increase risk of moderate to severe hematuria (14). Culkin DJ et al. in their review of anticoagulant therapy in urological practice, which included 79 articles, recommended that prostate biopsy is safe in patients on low dose aspirin with a risk of minor bleeding approximately three times higher than in controls (15).

In our study, we did not observe significant difference in number of hematuria episodes, number of patient reporting hematuria, rectal bleeding and hematospermia among the two groups. None of the patients required intervention for their hemorrhagic complication. Our result conform to some studies reported in literature (3, 8).

Limitations of the study include: a) non randomized nature and; b) follow-up limited to 3 weeks. The prospective randomized study among TRUS guided prostate biopsy patients taking low dose aspirin may be required for definitive conclusion.

## CONCLUSIONS

Continuing low dose aspirin during TRUS guided prostate biopsy neither alters the minor bleeding episodes nor causes major bleeding complications. So, discontinuation of low dose aspirin prior to TRUS guided prostate biopsy is not required.
